# Application and evaluation of nucleic acid sequence-based amplification, PCR and cryptococcal antigen test for diagnosis of cryptococcosis

**DOI:** 10.1186/s12879-021-06678-4

**Published:** 2021-09-29

**Authors:** Yanping Wang, Mi Yang, Yun Xia, Jia Yan, Jiaqi Zou, Dawei Zhang

**Affiliations:** 1 Department of Laboratory Medicine, The First Affiliated Hospital of Chongqing Medical University, 1 You Yi Road, Yuzhong District, Chongqing, 400016 China; 2 Department of Clinical Laboratory, Chengdu Women’s and Children’s Central Hospital, School of Medicine, University of Electronic Science and Technology of China, Chengdu, 611731 China

**Keywords:** NASBA, PCR, Capsular polysaccharide antigen, Cryptococcosis

## Abstract

**Background:**

Cryptococcosis is a major opportunistic invasive mycosis in immunocompromised patients, but it is also increasingly seen in immunocompetent patients. In the early stages of cryptococcosis, limitations of the detection method may hinder the diagnosis. A molecular diagnostic technique based on nucleic acid sequence-based amplification (NASBA) method was developed to fulfil the need for efficient diagnosis of cryptococcosis.

**Methods:**

We compared the diagnostic performance of NASBA, PCR and cryptococcal antigen (CrAg) test (colloidal gold method) in clinical samples from 25 cryptococcosis patients (including 8 cryptococcal meningoencephalitis and 17 pulmonary cryptococcosis) who were categorized as proven cases (n = 10) and probable cases (n = 15) according to the revised EORTC/MSG definitions. 10 patients with non-Cryptococcus infection and 30 healthy individuals were categorized as control group.

**Results:**

The lowest detection limit of NASBA was 10 CFU/mL, and RNA of non-target bacteria or fungi was not amplified. The sensitivity of NASBA, PCR and colloidal gold method was 92.00% (95% CI 72.50–98.60%), 64.00% (95% CI 42.62–81.29%), 100.00% (95% CI 83.42–100.00%), and the specificity was 95.00% (95% CI 81.79–99.13%), 80.00% (95% CI 63.86–90.39%) and 82.50% (95% CI 66.64–92.11%) respectively. The highest specificity (97.50%), accuracy (95.38%) and k value (0.90) were achieved when both NASBA and colloidal gold results were positive.

**Conclusions:**

NASBA is a new alternative detection method for cryptococcosis which is both accurate and rapid without expensive equipment and specialised personnel. It may be used as a tool for confirming current infection as well as monitoring the effectiveness of antifungal treatment. The use of NASBA to detect *Cryptococcus* RNA in blood samples is of great significance for the diagnosis of pulmonary cryptococcosis. The combination of NASBA and colloidal gold can improve the diagnostic accuracy of cryptococcosis.

## Background

Cryptococcosis is a fungal infection caused by encapsulated yeasts of the phylum Basidiomycota, genus *Cryptococcus*. *Cryptococcus neoformans* (*C. neoformans*) and *Cryptococcus gattii* cause the majority of cryptococcal infections in immunocompromised patients, resulting in varying complications [1]. But there are also reports showing that pulmonary cryptococcosis occurs more frequently in immunocompetent patients than in immunocompromised ones [2]. According to the revised definitions of invasive fungal disease from the European Organization for Research and Treatment of Cancer/Invasive Fungal Infections Cooperative Group and the National Institute of Allergy and Infectious Diseases Mycoses Study Group (EORTC/MSG) Consensus Group [3], laboratory tests such as culture, microscopic analysis and antigen detection, are of great significance for diagnosis of proven or probable cryptococcosis, especially for proven cryptococcal meningoencephalitis. Although isolation of the fungus from cultured tissue or body fluids (sputum, blood and cerebrospinal fluid) is the most commonly used detecting method in laboratories, it is relatively time-consuming for identifying the microorganisms [4]. In fact, direct microscopic examination is rapid, but the sensitivity is very low even if India ink staining is used [5], and fungal structures are only observed when the infection is advanced [6]. The detection of CrAg by colloidal gold method is one of the most helpful fungal routine tests, which is easy to use and has better sensitivity than other conventional methods for rapid diagnosis of early *Cryptococcus* infection [7, 8]. However, false-positive as well as false-negative results have been caused because of many factors such as rheumatoid factor, cross antigens of other fungi or bacterial and unknown non-specific proteins. Moreover, it is also unreliable for monitoring the efficacy of antifungal treatment [9, 10].

Recently, the diagnostic limitations and increasing incidence of fungal infections have prompted the development of tools for rapid and accurate diagnosis by using molecular methods based on nucleic acid amplification, including Polymerase Chain Reaction (PCR) and Nucleic acid sequence-based amplification (NASBA). Although in previous studies, PCR-based molecular methods provided both high sensitivity and specificity, the results were susceptible to DNA contamination [11]. NASBA assay has shown great potential in clinical application and has been successfully applied to detect bacteria, viruses, molds and parasites [12–15]. Compared to PCR, NASBA has a higher amplification efficiency because it can yield more than 10^12^ amplicons in as little as 30 min [16–18], which only takes about 2 h to perform each assay. Therefore, even if only a small amount of cryptococcal nucleic acid has been released from the fungal infection into blood or CSF, rapid amplification by NASBA can increase the positive rate. In our previous studies, the NASBA assay described by Park C has been successfully validated in diagnosing invasive aspergillosis [19–21]. However, the use of NASBA for cryptococcal diseases has not been described so far. The aim of this study was to develop a NASBA system to detect *Cryptococcus* RNA in serum or cerebrospinal fluid (CSF) for rapid diagnosis of cryptococcal infection.

## Methods

### Fungal and bacterial strains

*Cryptococcusneoformans* FY226, isolated from clinical samples, was cultured on blood agar at 37 °C for 1–2 days. It was identified as *C. neoformans* by ITS gene sequencing [22]. Spores of *Cryptococcus* were diluted with saline and quantified using a Neubauer chamber. A solution containing approximately 10^5^ spores was used for total RNA extraction. To assess the specificity of NASBA, the following strains of fungi and bacteria were used: *Aspergillus fumigatus* (*A. fumigatus* CMCCA1a), *Fusarium moniliforme* (*F. moniliforme* ATCC MYA-3629), *Candida albicans* (*C. albicans* ATCC 64548), *Candida parapsilosis* (*C. parapsilosis* ATCC 22019), *Staphylococcus aureus* (*S. aureus* ATCC 29213), *Pseudomonas aeruginosa* (*P. aeruginosa* ATCC 27853), *Escherichia coli* (*E. coli* ATCC 25922). All of the strains were cultured on blood agar at 37 °C for 1–2 days and the total RNA was extracted with Trizol reagent (Invitrogen, California, America) strictly following the Manufacturer’s instructions.

### Patient populations and clinical samples

A total of 65 inpatients were enrolled from 2017 to 2019 in the First Affiliated Hospital of Chongqing Medical University and categorized as proven cases (n = 10), probable cases (n = 15) and non-cryptococcosis (n = 40) according to the revised EORTC/MSG criteria. A proven cryptococcal meningoencephalitis is based on positive CrAg in CSF or a positive result of an India ink preparation of CSF. To define as a proven pulmonary cryptococcosis, it is required that yeasts cells or spores were detected by histological analysis or culture of a tissue specimen which was taken from the infected site of lungs by a sterile procedure, or blood culture is positive. Probable pulmonary cryptococcosis requires the presence of a host factor, a clinical criterion, and symptoms consistent with the disease entity, and mycological evidence (encapsulated budding yeasts cells or spores in bronchial alveolar lavage or sputum be detected by India ink staining or in the culture, or *Cryptococcus* antigen test in bronchial alveolar lavage, sputum, serum be positive) [23]. Of the 10 proven cases, there were 8 cryptococcal meningoencephalitis and 2 pulmonary cryptococcosis while 15 probable cases were all pulmonary cryptococcosis. 10 patients with non-Cryptococcus infection (including two tuberculosis, two bacterial infections, three fungal infections other than *Cryptococcus* and three autoimmune diseases) and 30 healthy individuals without any clinical symptoms of bacterial, fungal or parasitic infection and chronic diseases were categorized as control group. Proven and probable cases were selected on the solid basis of sample availability and diagnosis. For patients with cryptococcal meningoencephalitis, the collected samples were CSF, while for patients with pulmonary cryptococcosis and control group, the collected samples were serum. Serum or CSF specimen were all collected prior to any antifungal agents were used and stored at – 80 °C.

### *Cryptococcus* RNA extraction

In order to determine the analytical sensitivity of NASBA assays, spore suspensions of *C. neoformans* FY226 with a certain concentration (106 CFU/mL, 105 CFU/mL, 104 CFU/mL) were prepared by counting with a microscope. 100 μL of fungal suspension ground in mortar (free of Rnase) with liquid nitrogen was used for RNA extraction by a total RNA rapid extraction kit (Trizol) according to our previous study [21]. For samples of clinical patients, total RNA was extracted from serum or CSF using a blood/liquid sample total RNA rapid extraction kit (BioTeke, Beijing, China). In short, 250 μL of serum or CSF and 750 μL of RLS lysis buffer were mixed in a 1.5 mL microcentrifuge tube and incubated at room temperature for 10 min. Then 150 μL of chloroform was added, and the tube was vigorously vortexed for 15 s followed by incubation at room temperature for 3 min. After the mixture was centrifuged at 12,000×*g* for 10 min at 4 °C, the aqueous phase was transferred to the spin column AC and mixed with 600 μL 70% ethanol, followed by sequential washing of the column with buffer RE and buffer RW. Finally, RNA was eluted with 30 μL RNase-free water and stored at − 80 °C for further use.

### Primers

A highly conserved capsular-associated protein (CAP10) region specific to *Cryptococcus* genus was chosen as the detection target, and the primer sequence was designed by bioinformatics method according to previous reports (P1:5ʹ-AATTCTAATACGACTCACTATAGGGCCAAGCCCCCAAACCTCCCATAC-3ʹ; P2:5ʹ-AACGCGTACCATTCATCAAAGCC-3ʹ) [24]. This pair of primers was used to amplify a 229 nucleotide fragment of the target RNA.

### NASBA assay of cultured clinical strains and clinical samples

For NASBA reaction, *Cryptococcus* RNA sample (5 μL) was suspended in 10 μL of amplification buffer reagent consisting of 2 mmol/L each NTP (Takara Bio), 70 mmol/L KCl, 40 mmol/L Tris–HCl (pH 8.5), 12 mmol/L MgCl_2_, 5 mmol/L dithiothreitol, 0.375 mol/L sorbitol, 0.4 mmol/L each primer, and 12 U ribonuclease inhibitor (Promega, Fitchburg, WI, USA) in 10% dimethyl sulfoxide. The mixture was incubated at 65 °C for 5 min, then the primer was annealed at 41 °C for 5 min, followed by addition of 5 μL enzyme mixture (40 U T7 RNA polymerase (Promega), 0.1 U RNase H, 8 U avian myeloblastosis virus reverse transcriptase (Takara Bio) and 2.0 μg bovine serum albumin) [25]. The final mixture was amplified isothermally at 41 °C for 90 min, and the amplification products were analyzed by 1% agarose gel electrophoresis. To detect any contamination, an equal volume of RNase-free water was used as a negative control and RNA extracted from *C. neoformans* in pure culture was used as a positive control in all NASBA assays.

### DNA extraction and PCR assay

DNA was extracted from serum or CSF using a QIAamp blood/liquid mini kit (Qiagen, Hilden, Germany) according to the manufacturer’s instructions. Briefly, 200 μL of serum or CSF was mixed with 20 μL protease solution in a microcentrifuge tube and the tube was vigorously vortexed for 15 s after addition of 200 μL AL buffer, followed by incubation at 56 °C for 10 min. Then 200 μL absolute ethanol was added to the mixture and transferred to a QIAamp mini spin column. After centrifugation of the column at 6000×*g* for 1 min, the filtrate was discarded and the column was washed sequentially with 500 μL buffer AW1, 500 μL buffer AW2 as well as 200 μL buffer AE. Finally, DNA was eluted with 40 μL buffer AE and stored at − 20 °C. Purified DNA was amplified by PCR assay using previously reported primers targeting the CAP10 gene (P1:5ʹ-CCAAGCCCCCAAACCTCCCATAC-3ʹ, P2:5ʹ-AACGCGTACCATTCATCAAAGCC-3ʹ) [24]. The PCR system consisted of 3 μL of purified DNA in a total PCR volume of 25 μL with 1.0 μL each primer, 12.5 μL Taq polymerase (Takara Bio) and 7.5 μL sterile pure water. The mixture was incubated at 94 °C for 5 min, 35 cycles of 45 s at 94 °C, followed by 56 °C for 45 s and 72 °C for another 45 s, and the final extension was at 72 °C for 7 min. Negative control (template as an equal volume of pure water) and positive control (DNA extracted from *C. neoformans* in pure culture) were also included in each run.

### Cryptococcal capsular polysaccharide antigen detection

Colloidal gold kits (IMMY, Norman, OK) were used to detect *Cryptococcus* capsular polysaccharide antigen in clinical samples of this study according to the manufacturer’s recommendations. The whole blood sterile samples or CSF were collected by routine aseptic method. After serum and CSF supernatant were separated, 40 μL of sample dilution was added into a small test tube and mixed with 40 μL serum or CSF supernatant. Finally, a test strip was inserted into the mixture and the result was read after 10 min. The result was negative if there was only the presence of quality control line on the test strip. If there were both quality control line and test line, the result was judged as positive, and only the test line indicated an invalid test.

### Statistical analysis

IBM SPSS Statistics software package version 22.0 (IBM, Armonk, NY) was used to evaluate the performance parameters of each assay through the construction of 2 × 2 tables. To calculate the sensitivity of these three methods, positive results from patients with proven or probable cryptococcosis based on the EORTC/MSG criteria were served to be true positive. For calculation of the specificity, negative results from patients without evidence of cryptococcosis according to the EORTC/MSG criteria were considered to be true negative. The sensitivity, specificity, positive predictive value (PPV), negative predictive value (NPV), positive likelihood ratio, negative likelihood ratio, and accuracy were calculated for each assay. The comparison between two methods was performed by paired diagnosis test design using McNemar or Fisher’s exact test with a *P* value of ≤ 0.05 being considered significant. The Youden index was calculated to assess the ability of each assay to identify real patients and non-patients. The k statistic was applied to evaluate the consistency of the three methods [26] and interpreted as follows: excellent agreement between tests, k > 0.80; substantial agreement, 0.60 < k ≤ 0.80; moderate agreement, 0.40 < k ≤ 0.60; and poor agreement, k ≤ 0.40.

## Results

### Analytical specificity of NASBA

**Fig. 1:**
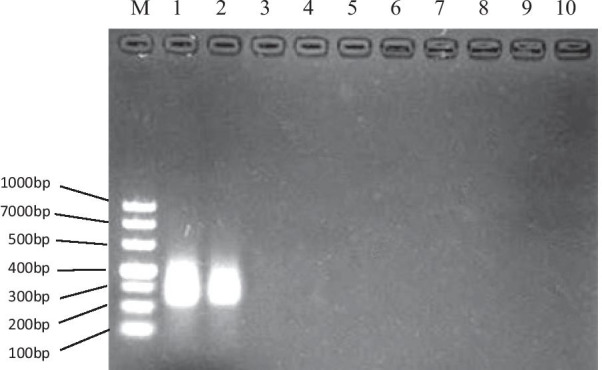
Electrophoresis results of NASBA products from different strains. M: DL1000bp DNA marker; 1–2: *C. neoformans* (FY 226); 3: *A. fumigatus* (CMCCA1a); 4: *F. moniliforme* (ATCC MYA-3629); 5: *C. albicans* (ATCC 64548); 6: *C. parapsilosis* (ATCC 22019); 7: *S. aureus* (ATCC 29213); 8: *P. aeruginosa* (ATCC 27853); 9: *E. coli* (ATCC 25922); 10: Negative control

The positive result was a 229 bp fragment of cultured *C. neoformans* FY226, which matched the CAP10 gene of *C. neoformans* standard strain H99 best after sequencing and BLAST analysis in NCBI database. No positive results were demonstrated for control strains *A. fumigatus, F. moniliforme, C. albicans, C. parapsilosis, S. aureus, P. aeruginosa and E. coli* (Fig. 1).

### Analytical sensitivity of NASBA

**Fig. 2:**
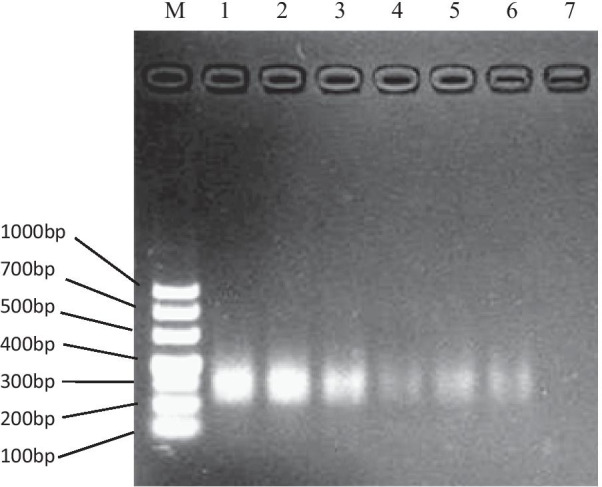
Electrophoresis analysis of NASBA products with gradient *C. neoformans* spores. M: DL1000bp DNA marker; 1–3: RNA extracted from the spore suspension of *C. neoformans* at a concentration of 10^6^ CFU/mL, 10^5^ CFU/mL, and 10^4^ CFU/mL, which was used as a template for NASBA; 4–7: RNA extracted from the spore suspension with a concentration of 10^4^ CFU/mL was diluted 10–10,000 times, and the concentrations corresponded to electrophoresis bands of 10^3^ CFU/mL, 10^2^ CFU/mL, 10^1^ CFU/mL, and 10^0^ CFU/mL, respectively

The analytical sensitivity of NASBA was acquired by using 1% agarose gel electrophoresis to analyze tenfold serial dilution of *C. neoformans* FY226 genomic RNA (Fig. 2). The lowest detection limit of NASBA was 10 CFU/mL after two repeated experiments.

### Comparison of individual assay performance

The results of 65 clinical specimens detected by NASBA, PCR and colloidal gold assays for CrAg were listed in Table 1. The NASBA results of 10 proven cases were all positive, and 13 of 15 probable cases were positive for it. The diagnostic parameters of sensitivity, specificity, accuracy, Kappa value, etc. were calculated for each assay (Table 2). The colloidal gold method had the highest sensitivity, while NASBA had the highest specificity among the three methods. Hypothesis testing of sensitivity revealed that NASBA and colloidal gold were significantly greater than that of PCR. There were no significant differences observed in sensitivity and specificity between NASBA and colloidal gold test. Overall, the NASBA assay performed best in accuracy, positive likelihood ratio, positive predictive value, Youden index, and Kappa value, while colloidal gold performed best in terms of negative likelihood ratio and negative predictive value.

**Table 1:** NASBA, PCR and colloidal gold results of clinical samples

EORTC/MSG	NASBA		PCR		Colloidal gold		Total
	+	−	+	−	+	−	
Proven cases (n = 10)	10	0	7	3	10	0	10
Probable cases (n = 15)	13	2	9	6	15	0	15
Control group (n = 40)	2	38	8	32	7	33	40
Total	25	40	24	41	32	33	65

EORTC/MSG, European Organization for Research and Treatment of Cancer/Invasive Fungal Infections Cooperative Group and the National Institute of Allergy and Infectious Diseases Mycoses Study Group

**Table 2:** Clinical diagnostic performance of NASBA, PCR and colloidal gold

Parameter	NASBA	PCR	Colloidal gold
Sensitivity [% (95% CI)]	92.00 (72.50–98.60)	64.00 (42.62–81.29)*	**100.00 (83.42–100.00)**
Specificity [% (95% CI)]	**95.00 (81.79–99.13)**	80.00 (63.86–90.39)	82.50 (66.64–92.11)
Accuracy [%]	**93.85**	73.85	89.23
Positive likelihood ratio	**18.40 (4.74–71.39)**	3.20 (1.61–6.35)	5.71 (2.92–11.20)
Negative likelihood ratio	0.08 (0.02–0.32)	0.45 (0.26–0.77)	**0.00 (0.00-NaN)**
Positive predictive value [% (95% CI)]	**92.00 (72.50–98.60)**	66.67 (44.69–83.57)	78.13 (59.56–90.06)
Negative predictive value [% (95% CI)]	95.00 (81.79–99.13)	78.05(61.97–88.89)	**100.00 (87.02–100.00)**
Youden index	**0.87**	0.44	0.83
Kappa	**0.87**	0.44	0.78

Best performance values are highlighted in bold. *P < 0.05 versus NASBA, colloidal gold

### Comparison of combined assay performance

We further analyzed the diagnostic efficacy of combined detection of NASBA, PCR and colloidal gold for cryptococcal disease, the diagnostic parameters of sensitivity, specificity, accuracy, and Kappa value were calculated and results are shown in Table 3. When both NASBA and colloidal gold results were positive, the highest specificity, accuracy and k value were obtained.

**Table 3:** Combined diagnostic performance of NASBA, PCR or colloidal gold

Test combination	Proven/probable cases (n = 25)	Control group (n = 40)	Sensitivity (%)	Specificity (%)	Accuracy (%)	Kappa
NASBA+ or PCR+	23	8	92.00	80.00	84.62	0.69
NASBA+ and PCR+	16	1	64.00	97.50	84.62	0.65
NASBA+ or colloidal gold+	25	7	100.00	82.50	89.23	0.78
NASBA+ and colloidal gold+	23	1	92.00	**97.50**	**95.38**	**0.90**
PCR+ or colloidal gold+	25	13	100.00	67.50	80.00	0.62
PCR+ and colloidal gold+	16	1	64.00	97.50	84.62	0.65
NASBA+ or PCR+ or colloidal gold+	25	14	100.00	65.00	78.46	0.59
NASBA+, PCR+ and colloidal gold+	16	0	64.00	100.00	86.15	0.69

Best performance values are highlighted in bold

## Discussion

Cryptococcal disease, once rare, has now become a major fungal infection in immunocompromised population worldwide, mainly in HIV-infected patients and people undergoing immunosuppressive therapy as well as those with lymphoproliferative disorders [27]. Early diagnosis of cryptococcal infection is critical to improving clinical outcomes. The detection of CrAg can greatly increase the diagnosis rate of cryptococcosis, but it can also lead to some false positive results. And for pulmonary cryptococcosis, because of its various clinical manifestations, the diagnosis is relatively complex and easy to miss. In fact, the clinical symptoms of pulmonary cryptococcal infection are based on the observation of abnormalities in X-rays, but such findings are also related to Mycobacterium tuberculosis infection in patients with proven or suspected HIV infection [28]. Tuberculosis may overshadow other opportunistic infections, leading to delayed diagnosis and appropriate treatment [29]. Therefore, there is an urgent need for a new laboratory test other than CrAg as another important test method for the diagnosis of cryptococcosis.

Molecular diagnostics have been proven to be very promising for the diagnosis of fungal infection [30]. This is the first study to describe NASBA assay for the analysis of cryptococcal RNA in clinical samples. In general, 18S rRNA is used as a common amplification target in the NASBA process of invasive aspergillosis [20, 21]. However, CAP10 gene is considered to be a better target for the diagnosis of cryptococcosis because it can encode a highly conserved capsule-associated protein which is specific for the *Cryptococcus* genus and has become a most important factor determining the pathogenicity of *Cryptococcus neoforman*s [24]. In our report, the primers used in NASBA system were aligned in BLAST and ensured fine specificity, the specificity of NASBA was proved by the non-specific amplification signal when RNA of non-target bacteria or fungi was used as templates.

The lowest detection limit of NASBA was 10 CFU/mL, and the sensitivity of NASBA in clinical samples was significantly greater than that of PCR (*P* < 0.05). NASBA possesses a higher inherent amplification capability because each cDNA template produces numerous RNA copies in each cycle, while each cDNA merely doubles in number at each cycle of PCR. NASBA has comparable sensitivity to colloidal gold method and the results of 10 proven cases were all positive. At the same time, of the 8 proven patients with cryptococcal meningoencephalitis, 5 had positive CSF culture results and only 3 had positive India ink results. Of the 2 proven and 15 probable patients with pulmonary cryptococcosis, bronchial alveolar lavage or sputum culture results were all negative. Therefore, compared with India ink staining or culture, the positive rate of NASAB was much higher. There were no statistical differences in the specificity of NASBA, PCR, and colloidal gold due to the small sample size. However, the specificity of NASBA reaches 95%, suggesting that it was an important method for confirming the diagnosis of cryptococcosis. The high specificity of NASBA may be that NASBA only allows amplification of template nucleic acids with T7 promoter and the thermal denaturation is absent, which can avoid the potential risk of contamination by non-target microbial nucleic acid or homologous DNA [31]. Since NASBA had the best performance in accuracy, positive likelihood ratio, positive predictive value, Youden index and k value among the three methods, it may be used for confirming cryptococcosis cases in routine laboratories. However, NASBA requires high quality specimens since RNA is easily degraded. And compared with DNA extraction, the RNA extraction process is more complicated and mainly operated manually, which may limit its wide use in fungal detection.

Our study yielded negative results in two patients with probable pulmonary cryptococcosis. We traced the specimen and found that one sample has been stored at − 80 °C for more than 1 year, the RNA in the specimen may have been degraded. For another negative result, the reason was that the patient had received antifungal therapy before admission, the *Cryptococcus* spores had been killed by antifungal drugs and could not release free RNA into blood. In contrast, the capsular polysaccharide antigen has a relatively long metabolic time lasting for several months, so colloidal gold result remains positive. The positive result of antigen will continue for a long time even after successful treatment. While for NASBA, the positive result can quickly turn negative once antifungal drugs are used. Therefore, NASBA is of great significance for confirming current cryptococcal infections, and may be used to monitor the effectiveness of antifungal treatment. However, there is also a clear limitation to this test that patients on empiric therapy prior to testing would be missed, leading to false negative results.

In present study, colloidal gold is the most sensitive assay among the three methods, the sensitivity appears to be consistent with that reported by Antinori [32]. Nevertheless, the specificity of colloidal gold is only 82.50%. To our knowledge, the specificity of colloidal gold technique is decreased due to the possibility of false-positive results, and it has previously been reported that CrAg cross-reacts with antigens of some fungi or bacteria such as *Trichosporon beigelii* and *Ustilago maydis* [33, 34]. The colloidal gold yielded 7 positive results for 40 non-Cryptococcus infection samples in this study. We followed up 5 of them, the clinical diagnosis of these five individuals involved one tuberculosis, two fungal infections other than *Cryptococcus* and two cases of autoimmune diseases. These false-positive results are probably due to that the presence of corresponding antigen in serum cross-reacts with CrAg, pronase has not been used in samples may be another important reason [35]. Importantly, we should also be aware of the condition defined as asymptomatic cryptococcal antigenemia, in which individuals with no symptoms may have positive antigen tests. The revised EORTC/MSG criteria recommend that such individuals may need to be followed and require treatment [36]. The colloidal gold method performed best in terms of negative likelihood ratio and negative predictive value, indicating that colloidal gold assay is suitable for screening patients suspected of *Cryptococcus* infection.

Although PCR assay has been reported to be recommended for the diagnosis of cryptococcal infection [37], its sensitivity in current study was significantly lower than the other two methods, even lower than previous reports [38]. The difference may be caused by the type of specimen selected. Most of the previous research groups had chosen cerebrospinal fluid as research specimen, while the subjects in this study were mainly serum specimens. Due to the neurotropic properties of *Cryptococcus*, most of them spread to the central nervous system through lung, reach pia mater, grow and reproduce massively [39]. Therefore, a large amount of *Cryptococcus* is enriched in cerebrospinal fluid while only a small amount of free nucleic acid is released from the fungal infection into blood, which leads to its low positive rate.

Combining the detection results of NASBA, PCR and colloidal gold to further explore their diagnostic efficacy for cryptococcosis, we found that when both NASBA and colloidal gold results were positive, the highest specificity, accuracy and k value could be obtained without obvious reduction of sensitivity. The combination of NASBA and colloidal gold can improve the diagnostic accuracy of cryptococcosis as well as achieve a highest agreement with the EORTC/MSG criteria, which is more beneficial to the diagnosis of cryptococcosis.

## Conclusions

This is the first report to describe NASBA for the diagnosis of *Cryptococcus* infection. The results showed that NASBA had good sensitivity and high specificity (absence of cross-reactivity with other unrelated bacteria or fungi). It may not only be used as a useful tool for rapid and accurate diagnosis of cryptococcosis but also for monitoring the effectiveness of antifungal treatment. We do not recommend the use of CSF for detection of NASBA because antigen detection is sensitive enough for the diagnosis of cryptococcal meningoencephalitis. However, the use of NASBA to detect *Cryptococcus* RNA in blood samples is of great significance for the diagnosis of pulmonary cryptococcosis. NASBA is suitable for use in less developed laboratories because it can be performed without any expensive equipment and specialised personnel. The combination of NASBA and colloidal gold can improve the diagnostic accuracy and is particularly useful in specific clinical situation (The negative colloidal gold result has an exclusion value, while the positive NASBA result has a confirmed value).

## Limitations

Of course, there are some limitations of this study. First of all, this is a retrospective study with a small number of cases, we will further expand the sample size in subsequent studies. Secondly, we did not compare the cost and operation time of the three methods because compared with PCR or colloidal gold, NASBA is mainly operated manually without commercial kits and automated extraction platforms. Finally, we did not use a molecular beacon in the NASBA process, the detection of gel electrophoresis is cumbersome with a qualitative result, and weak positive results are not easy to observe.

## Data Availability

The datasets generated and analyzed during the current study are available from the corresponding author on reasonable request.

## Abbreviations

NASBA: Nucleic acid sequence-based amplification
EORTC/MSG: European Organization for Research and Treatment of Cancer/Invasive Fungal Infections Cooperative Group and the NIH National Institute of Allergy and Infectious Diseases Mycoses Study Group
CSF: Cerebral spinal fluid
*C. neoformans*: *Cryptococcus neoformans*
*A. fumigatus*: *Aspergillus* *fumigatus*
*F. moniliforme*: *Fusarium* *moniliforme*
*C. **albicans*: *Candida albicans*
*C. parapsilosis*: *Candida parapsilosis*
*S. aureus*: *Staphylococcus aureus*
*P. **aeruginosa*: *Pseudomonas aeruginosa*
*E. **coli*: *Escherichia coli*
PPV: Positive predictive value
NPV: Negative predictive value
